# Using Precision Teaching to Improve Typically Developing Student’s Mathematical Skills Via Teleconferencing

**DOI:** 10.1007/s10864-023-09520-w

**Published:** 2023-05-23

**Authors:** Geetika Kapoor, Athanasios Vostanis, Suzy Mejía-Buenaño, Peter E. Langdon

**Affiliations:** 1 EdEssential, Punjabi Bagh, New Delhi, India; 2 Tizard Centre, University of Kent, Cornwallis North East, Canterbury, Kent, CT2 7NF UK; 3 Centre for Educational Development, Appraisal and Research, University of Warwick, Coventry, CV4 8UW UK; 4 Coventry and Warwickshire Partnership NHS Trust, Coventry, CV6 6NY UK

**Keywords:** Standard celeration chart, Mathematics, Behavioral fluency, Telehealth, Mainstream education

## Abstract

This study evaluated the effects of Precision Teaching in improving typically developing students’ mathematical skills when delivered via teleconferencing in India. Four students received Precision Teaching, while nine acted as control participants. Precision teaching involved instruction in three mathematical skills; two prerequisite skills and the primary skill of mixed addition and subtraction facts. Instruction included untimed practice, timed practice, goal-setting, graphing, and a token economy. Participants who received Precision Teaching received ten practice sessions for the prerequisite skills and 55 sessions for the primary skill. The results demonstrated improvements in the prerequisite skills of varied magnitude and considerable improvements in the primary skill, which were maintained above baseline performance levels. In addition, those who received Precision Teaching were below the 15th percentile rank at the initial assessment and above the 65th percentile at the post-intervention assessment in the math fluency subtest of the Kaufman Test of Educational Achievement—Third Edition. Control participants did not demonstrate similar improvements. Results suggest that Precision Teaching could produce accelerated outcomes even when delivered via teleconferencing. Therefore, it could be a valuable system for helping students ameliorate potential learning losses resulting from the COVID-19 pandemic.

## Introduction

The recent COVID-19 pandemic led to major educational disruptions, with an abrupt shift to online learning, and adverse effects on student well-being and attainment (Chaabane et al., 2021; Spitzer & Musslick, 2021). Students lost access to various essential resources such as school-based health care, nutrition programs, and additional special education support (Chaabane et al., 2021). An analysis of the pandemic’s impact on students is currently underway, with emerging evidence suggesting a negative impact on academic performance, unevenly spread across students from different socioeconomic backgrounds, and across subjects, with a magnitude that is still unclear (Gore et al., 2021; Renaissance, 2022; Sass & Goldring, 2021; Weidmann et al., 2021). Specifically, the pandemic highlighted that students from disadvantaged backgrounds are more likely to experience more significant learning losses due to barriers that are not solely related to educational factors but also socioeconomic and cultural ones (Harmey & Moss, 2021). Along with the additional factors that have historically affected students’ attainment (e.g., the family’s economic status or the presence of chronic conditions; Naven et al., 2019), the pandemic also highlighted that mathematics was one of the most affected subjects. Despite the different methodologies used in published studies and reports, the data suggest considerable learning losses in this area (Gore et al., 2021; Weidmann et al., 2021). As a result, increasing attention has been placed on mitigating them, with the importance of achieving basic educational standards and mastering pre-requisite skills at the forefront (UNESCO, 2022).

In their attempt to meet those educational standards during the pandemic, teachers relied on the use of technology, including the use of video conferencing software such as Zoom or Microsoft Teams, digital platforms to upload content such as Google classroom or Moodle, educational software such as Kahoot! or Google Jamboard, digital libraries, and massive open online courses. These resources have been used differently, with teachers integrating them into synchronous and asynchronous teaching. However, the use of technology by itself does not necessarily mean that good quality provision is provided to students. There is still a need for precise learning objectives, evidence-based strategies that promote active student responding, and progress monitoring to guide decision-making (Barbetta & Morales, 2021; Hamilton et al., 2016). One system that typically integrates these components is the system of Precision Teaching (PT). This system focuses on precisely measuring performance changes across time and uses standardized visual displays to engage in dynamic and strategic decision-making to accelerate performance and learning (Evans et al., 2021).

Precision Teaching follows a five-step framework, namely *pinpoint, practice, chart, decide, and try again*. In the pinpoint phase, Precision Teachers identify the skills to be developed and use precise definitions that make possible the sensitive monitoring of students’ performance. Definitions are constructed by using *movement cycles* and *learning channels*. Movement cycles include an action verb and an object (e.g., Says digit or Writes word), are repeatable, and have a discrete beginning and end (Kubina & Yurich, 2012). Learning channels specify the modality of instruction. For example, in the See-Say channel, the students *See* the question and *Say* the answer (Haughton, 1980; Lindsley, 1980). In the practice phase, Precision Teachers arrange instruction relevant to each skill and tailored to the students’ learning stage (Jimenez et al., 2021). As a result, instruction typically incorporates activities focused not only on acquisition, but also fluency. In the charting phase, they use a family of standardized visual displays purposefully built to demonstrate current performance levels and learning rates across time in a way that highlights when improvements plateau or students regress. These visual displays are known as the Standard Celeration Charts (SCCs), and they are considered essential when engaging in PT (Calkin, 2005). In the decide phase, Precision Teachers evaluate the data typically plotted as separate data paths for correct and incorrect responses, creating what is known as a *learning picture.* Based on those pictures, they identify whether a skill has been mastered, needs more practice, or requires additional remedial action, such as using different activities or even working on other related skills (White & Haring, 1980). In the try again phase, Precision Teachers represent the instruction after it has been modified to meet each student’s needs better. Precision Teachers particularly focus on instructional arrangements to explain lack of progress instead of pathologizing the students. Therefore, they encourage them to try again to meet their educational aims.

Precision Teaching has been applied to various areas such as literacy (Datchuk et al., 2015; Sawyer et al., 2021), fine motor skills (Vascelli et al., 2020, 2022), and mathematics (Greene et al., 2018; McTiernan et al., 2018; Sleeman et al., 2021). Specifically, Greene et al. (2018) conducted a stratified randomized controlled trial to evaluate cross-age peer tutoring and fluency-based instruction embedded within a PT framework. Typically developing students aged 8–12 from a disadvantaged area were recruited and practiced addition and subtraction. Participants in the experimental group received a multi-component intervention, including peer tutoring on timed fluency-based activities, confirmatory and corrective feedback, and reward systems to maintain motivation. They also had folders with all the necessary datasheets, SCCs, progress charts, flashcards, and worksheets. After an 8-week intervention, participants in the experimental group made significant improvements in the Woodcock & Johnson III mathematics fluency subtest compared to the control participants. Similarly, McTiernan et al., 2018 conducted a parallel-group randomized controlled trial to evaluate fluency-based instruction embedded within a PT framework. Typically developing students with a mean age of 10 years were recruited and practiced addition and subtraction. In this case, as well, the intervention was multi-component and consisted of timed fluency-based activities, confirmatory or corrective feedback, and reward systems. Participants also had personalized folders with all the relevant materials, such as datasheets, SCCs, timers, and worksheets. After a 5-week intervention, participants made significant improvements in the Woodcock & Johnson III mathematics fluency subtest compared to the control participant. Sleeman et al. (2021) conducted a randomized controlled trial to evaluate the effect of a self-regulated learner framework using elements of *Detect, Practice, and Repair* combined with PT. Detect, Practice, and Repair is a set of procedures focused on improving students’ mathematical skills (Poncy et al., 2013). In the Detect stage, students’ ability to answer math facts at a natural pace is assessed. That way, students’ dysfluency with specific math facts is highlighted. During the Practice stage, the first several uncompleted math facts are practiced using the Cover, Copy, and Compare instructional strategy. That way, instruction is individualized (Poncy et al., 2010). In the Repair stage, a timed assessment is conducted using an alternate form to the one used in the Detect stage. Students’ performance is measured and typically graphed by the students. That way, performance improvements are highlighted (Poncy et al., 2013). In the study conducted by Sleeman et al. (2021), typically developing students aged 9–10 years were recruited from an above-average socioeconomic area and practiced basic multiplication facts. The multi-component intervention consisted of self-regulatory training, timed fluency-based activities, goal-setting and graphing, and resources, including flashcards and worksheets. Participants in the experimental group received the intervention for 15 min daily and made significant improvements compared to the control participants.

Despite the encouraging evidence regarding PT’s application to improve mathematical skills, no studies have evaluated its effect when delivered in an online format using teleconferencing. This would be timely as the pandemic experience demonstrated that technology has much potential to provide accessible instruction to students. In addition, delivering PT in an online format could prove to be a valuable strategy for students in remote areas who might not be able to access additional instruction. To that end, this study aimed to evaluate the effects of a Precision Teaching framework delivered, as supplementary instruction, in an online format on typically developing students reported to be falling behind academically in the area of mathematics.

## Method

### Participants and Setting

Four students were recruited and received the intervention. That way, a sufficient number of participants allowed us to demonstrate experimental control in line with single-case design logic (Kazdin, 2016). All of them were reported to be falling behind in mathematics by their teachers. Therefore, recruitment was purposive. Three students were male, and one was female (see Table 1). Participants attended two urban private mainstream schools in New Delhi, India. The schools follow the national education policy and accommodate students from grade 1 to grade 12. Nine students, who were the participants’ peers and were reported to be fluent in mathematics, were also recruited in the study and acted as a control group. Specifically, those participants were assessed at the beginning and end of the study and allowed us to guard against threats to internal validity, such as history and maturation. All participants receiving Precision Teaching joined the sessions online from their home settings. Parents were asked to provide a room in the house that would allow participants to engage in the practice with minimal distractions. All participants, including the ones receiving Precision Teaching and the Control participants, were Indian, had Hindi as their first language, were attending Grade 3, and were typically developing.

**Table 1:** Participants’ Scores on the Norm-Referenced Assessment Tools

Participants	Sex	Age	KTEA-3			TEMA-3
			Math Application Score	Math Computation Score	Math Composite Score	Standard Score
PT1^a^	Male	8:2	83	81	81	81
PT2	Female	7:9	93	103	98	89
PT3	Male	7:11	109	98	104	90
PT4	Male	8:0	87	105	95	87
C1^b^	Male	8:11	91	87	88	89
C2	Male	8:4	99	100	99	103
C3	Female	8:0	100	107	104	108
C4	Male	7:10	100	120	111	121
C5	Female	8:3	111	103	108	103
C6	Female	7:9	113	113	114	115
C7	Male	8:7	101	110	106	115
C8	Male	8:2	102	97	99	104
C9	Female	8:7	93	104	97	97

All participants were Indian, Hindi was their first language, had no diagnoses, and attended Grade 3. The assessments were conducted via Zoom to provide descriptive information about participants’ abilities. KTEA-3 = Kaufman Test of Educational Achievement—Third Edition; TEMA-3 = Test of Early Mathematics Ability-Third Edition

^a^PT1 to PT4 are the participants that received the intervention. ^b^C-1 to C-9 are the participants that received TAU conditions

### Eligibility Criteria

For inclusion in the study students needed to (a) be typically developing, (b) attending Grade 3, (c) have been identified by their teacher as falling behind academically, (d) have produced a standard score below 85 (i.e., more than one standard deviation away from the mean) on the math fluency subtest of the Kaufman Test of Educational Achievement—Third Edition (Kaufman & Kaufman, 2014), and (e) have access to an internet connection and a computer, laptop, or tablet. If students had an official diagnosis of an intellectual or developmental disability, they were excluded from the study. Once a favorable ethical opinion was received by the ethics of committee of the University of Kent in England and GD Goenka University in India, students were invited to take part in the study following parental consent to include them.

### Materials

#### Assessment Tools

Two assessment tools were used in this study, to guide participants’ selection and provided additional information about their mathematical ability (see Table 1). The first one was the Kaufman Test of Educational Achievement—Third Edition (KTEA-3; Kaufman & Kaufman, 2014), and the second one was the Test of Early Mathematics Ability—Third Edition (TEMA-3; Ginsburg & Baroody, 2003). KTEA-3 is a an individually administered battery for the assessment of key academic skills for individuals aged 4–25 years old. It thoroughly assesses reading, writing, oral language, and mathematical skills by using 19 subtests. The split-half reliability of its composites has been reported to range between the 0.80 s and 0.90 s, with the only exception being Oral Fluency at the 0.70 s. Similarly, the average split-half reliability of the subtests has been reported to be between the 0.80 s and 0.90 s, with the only exception being the Oral expression subtest ranging from the 0.60 s to the 0.90 s (Kaufman & Kaufman, 2014). TEMA-3 is a 72-item test measuring an individual’s mathematical ability, including (a) counting proficiency, (b) cardinality, (c) number comparison facility, and (d) elementary arithmetic (Ginsburg & Baroody, 2003). It has been normed in 1219 students, with and without disabilities attending mainstream education, aged from 3 to 8 years. Its internal consistency has been reported to be between 0.94 and 0.96 and test–retest reliability between 0.82 and 0.93 (Ginsburg & Baroody, 2003).

#### General Classroom Materials

The experimenter and Precision Teaching participants used computer-based resources including their laptops and smartphones, a video conferencing software called Zoom, a free multiplatform messaging app called WhatsApp, and the Google suite, which included Jamboard, Docs, Slides, Sheets, and Drive. Participants also used an online tally counter (www.scorecounter.com) and digital timers.

The experimenter used Zoom to conduct the training of Precision Teaching participants synchronously and WhatsApp to communicate with them and their families regarding session arrangements and their progress. They used Google Docs to create the participants’ points board. The board was sized 8.3 × 11.7 inches, in portrait orientation using a 12 Times New Roman font in black color. It had ten rows including information such as the participants’ name and the date. It also had a 6 × 4 table that the teacher used to provide points to the participant during the lesson. They used Google Slides to create a grid with available games that participants could exchange their points for at the end of the practice. Each option was displayed in a different cell of a 5 × 3 table created on one of the slides. They used Google Sheets to collect participants’ data and plot them on two different equal-interval graphs, namely a Timings and a Daily graph. Each graph was placed in a different page and had accompanying color-coded cells that acted as a datasheet. The cells related to the date, day, and number of timings were grey and were filled out in advance by the experimenter. The cells for correct responses were green, for incorrect red, and for skips yellow and were filled out during the session by the experimenter and each participant. The first graph was used to plot the scores of each timing, and the other was used to plot the best score of the day. The timings graph’s x-axis was divided into five days, and each day was divided into five timings, while the daily chart had successive calendar days on the x-axis. All graphs were pre-made so the experimenter could add the data, and the participants could automatically examine their progress. Although participants used equal-interval graphs, the authors plotted the data on the SCCs to monitor progress ongoingly. Optimally, the participants themselves would be charting their data on the SCCs. However, we decided to use simpler graphs with participants as there is no available software that provides the SCCs for free, and the focus of this study was to use as many accessible resources as possible. All resources were stored in the Google Drive cloud service and were accessible to the authors. The resources were shared with participants in a screen share mode, during the session timings. Therefore, participants and their families did not have editing rights to any of the resources used in this study, to ensure that the data were not accidentally changed. The online tally counter was used as a feedback tool during the practice sessions to allow students to monitor their performance during untimed practice.

The control group also used computer-based resources including their laptops and smartphones, Zoom, and WhatsApp. In this case, the experimenter only assessed participants’ ability and that is why some resources were not used including, Jamboard, Docs, Slides, Sheets, the Drive, or the online tally counter, as these were components of the Precision Training delivered.

#### Materials for Mathematical Practice

Worksheets were created by the authors, using Microsoft Excel and Word, and were randomly allocated to each participant by using a dedicated webpage at www.randomizer.org. The control participants only used the worksheets created for timed practice, specifically the ones including the review slice, to evaluate their overall performance pre- and post-intervention. The Precision Teaching participants used additional worksheets focused on timed and untimed practice during training. Specifically, for *See-Says number 0–20 randomly presented,* the worksheets randomly presented these numbers in four columns. Participants were expected to read the numbers on each column aloud before moving to the next one. For *See-Say counts three numbers (upward or downward) from a different starting number between 0 and 20,* the sheets randomly presented numbers between 0 and 20. Each number was followed by either an up- or down-facing arrow mark, signaling the student to vocally count up or count down the next three numbers, respectively. For both skills worksheets were divided into ones for untimed and timed practice. All worksheets were sized 8.3 × 11.7 inches, in landscape orientation using a 16 Times New Roman font in black color. For the first skill, the untimed worksheets had two columns of 11 numbers each for a total of 22 numbers per page. The timed worksheets had four columns of 11 numbers each for a total of 44 numbers per page. The untimed worksheets had fewer numbers per page to ensure that untimed practice would be brief. This decision was made as untimed practice was repeated before each timing, and we wanted to avoid participants finding the practice too tiresome. For the second skill, the untimed worksheets had three numbers per page, each with an arrow next to it. The timed worksheets had 11 numbers per page, each with an arrow next to it. In this case, as well, the untimed worksheets had fewer numbers per page to ensure untimed practice was brief. For both skills, each worksheet had a counter part that was used by the teacher to score the participants’ vocal responses. For *See-Says addition or subtraction fact randomly presented* the untimed practice involved two different Google Sheets pages and a blank Jamboard slide. The first Google Sheets page had ten addition/subtraction facts that the students were expected to vocally answer. The second page had ten fact families where one number was missing (e.g., 3–8), and which the participants were expected to vocally identify. In Jamboard, the experimenter wrote the number family (e.g., 2, 3, 5) on the slide and participants were instructed to say all four possible combinations (e.g., 2 + 3 = 5, 3 + 2 = 5, 5–2 = 3, and 5–3 = 2), while the teacher transcribed them on the slide. For the timed practice, different worksheets were created for each subset of the curriculum (i.e., slice). Each worksheet was sized 8.3 × 11.7 inches, in portrait orientation using a 16 Times New Roman font in black color. Each page had 17 addition/subtraction facts presented horizontally. In this case as well, counterparts were created for the teacher to score the participants’ vocal responses. Finally, all worksheets had more pages than the participants could possibly complete during their practice to ensure that no artificial ceilings were placed on their performance.

### Dependent Variable and Research Design

**Fig. 1:**
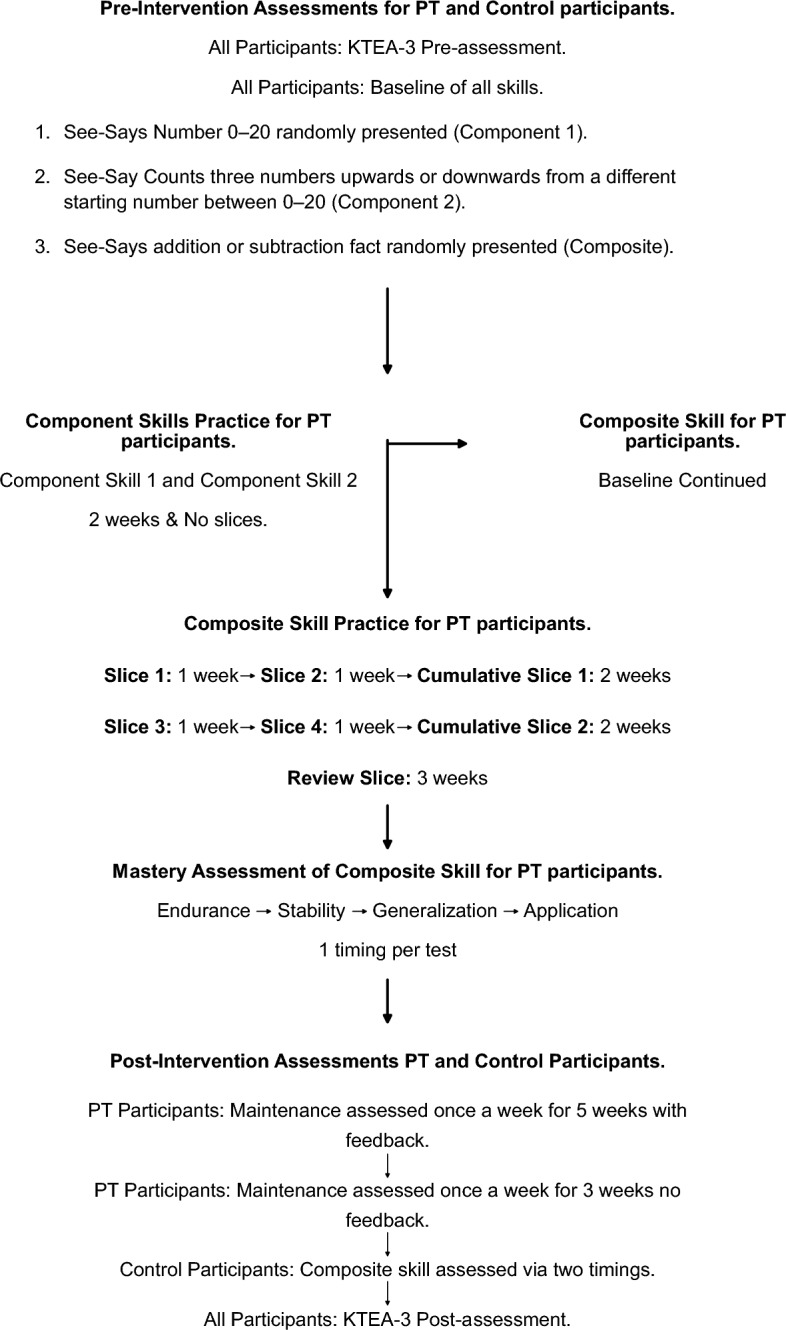
A sequence diagram of the study’s steps. PT participants = The participants who received the Precision Teaching intervention

We conducted a component-composite analysis (see Table 2) and identified a series of component skills (i.e., basic skills) that should be taught before the composite skill (i.e., complex skill). All these skills would typically be assessed and taught in educational settings before practicing the composite skill (for a detailed account, see Johnson et al., 2021; Stein et al., 2018). However, due to the timeframe of this study, the two most essential ones were chosen for practice in consultation with the participants’ teachers (see Fig. 1). Specifically, the same component skills were targeted across all participants for consistency and to protect the study’s internal validity. However, readers should note that this process should be individualized in applied practice. Each skill was pinpointed by using the learning channel matrix and movement cycles, following the PT framework (Haughton, 1980). The first skill was pinpointed as See-Says number 0–20 randomly presented and the second skill as See-Say counts three numbers (upward or downward) from a different starting number between 0 and 20.

**Table 2:** Example of a component-composite analysis

Component Skills	
See-Say counts object with 1:1 correspondence up to 10	See-Say counts pictorial representations with 1:1 correspondence up to 10
See-Says number 0–9	Free-Writes Number 0–9
See-Says number 0–100	See-Say identifies the plus, minus, and equal symbol
(Free-Say or Free-Write) counts from 0 to 20 in ascending order	See-Write adds the missing symbol from equation (plus, minus, equal)
(Free-Say or Free-Write) counts from 20 to 0 in descending order	See-Say estimates number of items in a group
(Free-Say or Free-Write) counts from 0 to 100 in ascending order	See-Say identifies ones and tens of a number provided
(Free-Say or Free-Write) counts from 100 to 0 in descending order	See-Write aligns numbers based on their place value (ones & tens)
(Free-Say or Free-Write) counts from 0 to 100 by 10 s in ascending order	See-Say solves + 1 addition fact
(Free-Say or Free-Write) counts from 100 to 0 by 10 s in descending order	See-Say solves -1 subtraction fact
(Free-Say or Free-Write) counts from 0 to 100 by 2 s in ascending order	(Free-Say or Free-Write) counts from 100 to 0 by 2 s in descending order

This table provides an example of a component-composite analysis. Readers should note that this list of skills is not exhaustive. The component-composite analysis should be informed by participants’ instructional history, grade/age, and current level of ability. For a more detailed outline of component skills, we recommend (Johnson et al., 2021; Stein et al., 2018)

The composite skill was mixed addition and subtraction facts using number families. A number family includes three numbers that can be combined in four different ways. For example, numbers 2, 3, and 5 are one number family and can produce four combinations, namely 2 + 3 = 5, 3 + 2 = 5, 5–3 = 2, and 5–2 = 3. For this study we included the number families for numbers 2, 3, 4, and 5. Each family combined numbers 2–9. Therefore, the combinations ranged from 2, 2, 4 to 5, 9, 14. Number families were chosen as they reduce the number of facts that need to be memorized by 75% (Johnson & Street, 2013). The composite skill was pinpointed as See-Says addition or subtraction fact randomly presented. The number of correct, incorrect, skipped digits per minute was recorded. The intervention effects were evaluated using a concurrent multiple baseline across participants design for each skill (Carr, 2005). The order with which participants were introduced to the intervention was randomly decided using www.randomizer.org.

### Curriculum Slicing

Curriculum slicing is typically used in Precision Teaching. During this process, a skill is broken into smaller subsets to make practice more achievable (Kubina & Yurich, 2012). For example, instead of teaching a whole multiplication table, Precision Teachers would typically break it into smaller slices (i.e., subsets) and train each to fluency before bringing them together. Slice 1 would include the first half of the table presented in random order, Slice 2 the second half, and the Review slice would consist of all the multiplication facts presented in a random order to avoid rote learning.

The two prerequisite skills were not broken into smaller slices (i.e., subsets) and were practiced as a whole for two weeks. We made this decision as the skills were not particularly complex. The composite skill was broken into seven slices as there were many fact families that participants had to memorize. Readers should note that Morningside Academy recommends 16 slices for this skill (Johnson et al., 2021). However, we decided to condense them to ensure that participants had an opportunity to practice all relevant math facts within the study’s timeframes. As a result, Slice 1 included families 2–2-4 to 2–9-11, slice 2 included families 3–2-5 to 3–9-12, cumulative slice 1 combined them and included families 2–2-4 to 3–9-12, slice 3 included families 4–2-6 to 4–9-13, slice 4 included families 5–2-7 to 5–9-14, cumulative slice 2 combined them and included families from 4–2-6 to 5–9-14, while the review slice included all the combinations from 2–2-4 to 5–9-14 (see Table 3). All individual slices were practiced for one week, each of the two cumulative slices was practiced for two weeks, and the review slice was practiced for three weeks. Thus, participants received a total of 11 weeks of practice on the composite skill. A pre-determined duration of practice was set for internal validity purposes (Ledford et al., 2022). In applied practice, the decision to proceed to the next phase of the instruction would be based on whether students have achieved fluent responding in a particular skill or slice, which is an approach we strongly encourage for optimal outcomes. However, for this study, it was essential to maintain the intervention’s consistency to compare performance gains more accurately among the Precision Teaching participants. To that end, instruction was offered for the same amount of time to all participants. Potentially, if this process had been more individualized, it would have led to even greater outcomes.

**Table 3:** An outline of the number families included in each slice of the composite skill

Slice 1	Slice 2	Cumulative Slice 1	Slice 3	Slice 4	Cumulative Slice 2	Review Slice
2 2 4	3 2 5	Slice 1 and Slice 2 combined	4 2 6	5 2 7	Slice 3 and Slice 4 combined	Slices 1 to 4 combined
2 3 5	3 3 6		4 3 7	5 3 8		
2 4 6	3 4 7		4 4 8	5 4 9		
2 5 7	3 5 8		4 5 9	5 5 10		
2 6 8	3 6 9		4 6 10	5 6 11		
2 7 9	3 7 10		4 7 11	5 7 12		
2 8 10	3 8 11		4 8 12	5 8 13		
2 9 11	3 9 12		4 9 13	5 9 14		

Each number family created four combinations. For example, 5 2 7 included 5 + 2 = 7, 2 + 5 = 7, 7–5 = 2, and 7–2 = 5

### Procedure

#### Practice Elements

This study utilized a multi-component intervention including (a) a component-composite analysis, (b) untimed practice, (c) timed practice, (d) goal setting, (e) graphing, and (f) a token economy in the form of a points board. The lessons were delivered by the first author who was a certified school psychologist and a board-certified behavior analyst (BCBA) with 12 years of experience. Throughout the duration of the Precision Teaching practice, control participants received Teaching as Usual in line with the national curriculum of India and the typical strategies used within their school settings.

#### Baseline

Baseline data were collected for at least five days, while some Precision Teaching participants received more baseline sessions following the conventions of the multiple baseline design. Each of the three skills was assessed by asking participants to engage in a 1-min timing using the review slice, which included all the possible mathematical combinations. No practice or feedback was provided. Participants were praised for their participation and provided with 5-min’ worth of playtime. The control participants were also baselined once at the beginning of the study by following the same procedure. Due to the fact that this group of students was only assessed once, we allowed them to engage in two timings for each skill, which was the only difference in the way their performance was baselined. That way it was more likely that their performance would not be confounded by the novelty of the procedures. To that end, the control participants’ best score was used in the analysis.

#### Component Skills Practice

Precision Teaching participants practiced the component skills simultaneously. Once they completed their practice, they were introduced to the composite skill. Therefore, participants needed to complete practice with the component skills to be able to practice the composite skill.

At the beginning of the first week of practice with the component skills, participants engaged in two timings. Based on their best score, out of the two timings, the day’s performance criterion was calculated by increasing their score by one response.

Each lesson started by asking participants to fill out their points board and choose a preferred activity they would engage in at the end of the lesson. For each pinpointed skill the participants completed untimed practice, then timed practice, then graphed their performance, evaluated it against the performance criterion set, and either completed their practice or repeated the process. Untimed practice was different for each skill but followed the same process where the teacher engaged in the skill along with the participants for one page of each untimed worksheet and then provided feedback. For See-Says number 0–20, the worksheet was made available via screensharing, and the teacher and participants read aloud, in unison, one practice page of numbers. Similarly, for See-Say counts three numbers, the teacher and participants counted up or down, in unison, two practice pages of different starting numbers. For both skills and to avoid rote responding, the teacher varied the pages used for each practice. As participants familiarized themselves with the process the teacher faded out their involvement and only transcribed participants’ answers allowing them to complete the untimed practice independently.

During the timed practice, participants were asked to perform to the best of their ability, for one minute, without being interrupted. Once the timing was completed, they received confirmatory or corrective feedback and the teacher calculated their correct, incorrect, and skipped responses, and plotted their data on the timings chart. Confirmatory feedback included vocal praise and points delivered on the students’ board on a variable schedule of reinforcement (VR3). Points were delivered to participants for completing their untimed or timed practice, asking questions related to the lesson, or engaging in additional practice while receiving corrective feedback. Corrective feedback included revisiting incorrect responses, asking the student to state the correct response, and providing a few additional examples to provide an opportunity for independent responding. If participants met their daily criterion, practice was completed. Otherwise, they engaged in another round of untimed and then timed practice for a maximum of five timings. Potentially, students could have engaged in additional timings to increase their exposure to practice, which is quite common in Precision Teaching classrooms. However, we decided to keep the number at five to ensure that practice was as efficient as possible and to avoid prolonged exposure to mobile, tablet, or computer screens. When practice on a certain skill was completed, the teacher graphed their best score of the day on the daily graph, praised them, and provided a point on their board. Every time participants met the day’s criterion, it was raised by one response following the personal best goal-setting approach (Vostanis et al., 2021). If a participant did not meet the day’s criterion, it stayed the same for the next day. Upon the completion of the day’s practice the teacher prompted the students to count and exchange their points for their chosen activity. Activities included a variety of games such as word memory games, an in-house scavenger hunt, or solving riddles, to name a few.

#### Composite Skill Practice

As soon as practice on the component skills was completed, the composite skill was introduced. The process was the same the one described above, and the only difference was on the way the untimed practice was conducted. Untimed practice was conducted in three steps. First, the teacher presented the first Google sheets page with the randomized facts from the target slice, and participants were expected to vocally answer all ten of them, while the experimenter typed the answers in the sheet and provided confirmatory or corrective feedback. The teacher also used a visual tally counter, on the shared screen, to help participants monitor their performance. Second, the teacher wrote the three numbers of two number families on Jamboard (e.g., 2 2 4 and 2 4 6). The teacher and participants said, in unison, all the possible combinations, while the teacher transcribed the numbers on Jamboard. Third, the teacher presented the second Google Sheets page with ten randomized fact families where one number was missing in a random fashion (e.g., 3___8 for the fact family of 3, 5, 8). The teacher asked the participants to say the corresponding math fact and the number missing in each of the ten examples, (e.g., 8 minus 3 equals 5), while typing the answers for them and providing feedback. The teacher also selected two to three examples where she prompted the participant to say all the remaining combinations of that fact family. In this case as well, the teacher varied the number families and the missing numbers to avoid rote responding and offer multiple examples to students. Also, as soon as participants familiarized themselves with the process, the experimenter only transcribed their answers and allowed them to practice independently.

As soon as the participants engaged in a round of untimed practice, they participated in timed practice that was the same as the one described for the component skills. In this case, as well, the teacher screen-shared the worksheet and participants vocally answered the math facts. The teacher scored their responses by using a counterpart of the worksheet which included the answers.

#### Assessment of Mastery

When Precision Teaching participants completed their practice with the composite skill an assessment of endurance, stability, generalization, application, and maintenance (ESGA-M) was conducted following the guidelines by (Fabrizio & Moors, 2003). The ESGA assessment was conducted at the end of the last day of the composite skills practice. For the assessment of endurance participants completed a 3-min timing on the composite skill. For the assessment of stability participants engaged in a 1-min timing while the teacher tried to distract them by playing a number name video on YouTube. For the generalization assessment participants were asked to complete a 1-min timing using a novel worksheet that they have not used before that presented facts vertically. For the application assessment, participants worked on the Hear-Say learning channel set. The teacher read aloud math word problems, while the worksheet was visible on a shared screen and participants had to say their answers. Finally, the maintenance assessment was conducted in two phases, using two 1-min timings once a week. Participants were allowed to engage in two timings as the first timing possibly acting as a warm-up. The best score of the two timings was taken as their performance for that day. In the first phase Precision Teaching participants were assessed once a week for a total of five weeks, where they received feedback about their incorrect responses. In the second phase they repeated the process once a week for three weeks but did not receive any feedback about their performance. Therefore, the maintenance assessments lasted 8 weeks in total. Finally, they were assessed again with KTEA-3, which made possible the evaluation of their progress in relation to normative standards.

In the final week of the maintenance assessment, the control participants were also assessed again to allow us to examine their progress since their first baseline assessment at the beginning of the study. The procedure involved two assessments. First, the participants engaged in two timings on the composite skill’s review slice without receiving any feedback. Second, they were assessed again with KTEA-3, which allowed us to examine their general progress since the study’s initiation and in relation to normative standards.

### Performance Criteria

Performance criteria are widely used in precision teaching and are typically reported as a range of performance frequencies (e.g., 100–150 correct responses per minute). Students receiving Precision Teaching are expected to practice toward these criteria set as they are more likely to lead to fluent responding (Binder, 1996). In this study, performance criteria were highlighted on the Precision Teaching participants’ graph to show them what their ultimate performance should be and motivate them. The criteria were set based on the recommended aims in the field of Precision Teaching and specifically Morningside Academy (Johnson & Street, 2013). For the two component skills the aim was set at 180–200 correct digits said per minute with no more than two incorrect/skips. For the composite skill, the Morningside curriculum sets three levels of performance aims depending on the complexity of the slice being practiced. Specifically, 50–60 digits correct per minute for the simple slice, 60–70 for the cumulative slice, and 70–80 for the review (Johnson, 2008). To ensure that participants were as competent as possible, we set the highest performance criterion, which was 70–80 correct digits said per minute with no more than two incorrect/skips. If participants reached the high end of a performance criterion, it stayed the same for the remaining days of practice. That way participants were not expected to perform above the range of each performance criterion. This decision was taken to ensure that participants would not feel pressured to perform at higher frequencies, which would be unnecessary and potentially aversive. However, this protocol was not used as none of the participants reached the high end of the criterion during the study.

### Duration of Sessions

Precision Teaching participants completed ten practice sessions for the component skills. The average duration was 39 min (range: 29–46 min) for PT1, 47 min (range: 23–68 min) for PT2, 52 min (range: 38–66 min) for PT3, 43 min (20–63 min) for PT4. They also completed 55 practice sessions for the composite skill. The average duration was 37 min (range: 12–83 min) for PT1, 35 min (range: 13–58 min) for PT2, 38 min (range: 13–93 min) for PT3, 35 min (range: 11–69 min) for PT4.

### Absence Protocol

From the outset of the study, a protocol was in place to account for any absence from lessons due to illness or other reasons. If Precision Teaching participants missed one or two days of practice, then they practiced during the weekend to catch up. If they missed three days of lessons, then they restarted their weekly practice once they were available. PT1 practiced in the weekend one time during component skills practice and three times during composite skill practice. PT2 and PT3 practiced in the weekend three times during the composite skill practice, while PT4 practiced four times.

### Accuracy Assessment

All sessions were video recorded and as a result produced true values that made possible the data collection’s accuracy assessment. Specifically, the first author scored randomly chosen video-recordings of the sessions and scanned worksheets. Accuracy was calculated for at least 20% of the total number of sessions across all participants, skills, and slices. Specifically, for the component skills an average of 22.36% was scored, (range: 20%–24.44%), across the baseline and intervention sessions for all participants. For the composite skill an average of 30.87% was scored, (range: 30.44%–31.11%), across the baseline, intervention, ESGA, and maintenance sessions for all participants. Accuracy was calculated in two steps. First, agreement on correct digits, incorrect digits, and skipped facts was calculated separately by dividing the smaller by the larger number and then multiplying by 100. The three percentages were then added together and divided by three to produce the overall agreement for each skill. Second, the average overall agreement was calculated by including the agreement for each skill and from each phase including baseline, each slice practiced, and the ESGA-M assessment. The overall component skills accuracy for PT-1 was 100%, for PT-2 100%, for PT-3 98% (range: 97%–100%), and for PT-4 97% (range: 95%–100%). The overall composite skills agreement was 100% for all participants.

### Procedural Fidelity

The second author, a qualified teacher and doctoral level BCBA with thirteen years of experience, scored fidelity checklists by watching the same videos provided for interobserver agreement. Four sets of fidelity checklists were designed to correspond with each phase of the study, namely Baseline (13 items), Intervention (14 items), ESGA (8 items), and Maintenance (7 items). The first two applied to the component skills, while all four applied to the composite skill. The fidelity checklist included general items related to lesson’s setup and sequencing, while other items focused on the way timings were conducted. Each item was scored as Yes or No and a total percentage of implementation accuracy was calculated for each day. Fidelity was averaged across all phases of the study and was 99% (range: 86%–100%) for each participant. During the first maintenance fidelity assessments, it was noted that the experimenter accidentally provided feedback after each timing. To maintain consistency, we decided to provide feedback to participants for the first five weeks of the maintenance assessments and then added three weeks where no feedback was provided.

### Social Validity

Unfortunately, due to complications related to COVID-19, it was not possible to officially assess participants’ opinion about the study and had to rely on parental and teacher anecdotal reports provided, while the study was ongoing.

### Data Analysis

Data were plotted using an online software named PrecisionX, which provided the SCCs for visual analysis and calculated a series of behavioral metrics. Primary metrics utilized were level, celeration, bounce, and the level change multiplier. The level shows the average performance of the individual across time. The geometric mean was calculated as it is less affected by extreme variables (Everitt & Howell, 2005). Celeration (i.e., (count/unit of time)/unit of time) is a frequency-derived measure quantifying students’ learning rate across time. Celeration can be calculated across days, weeks, months, or even years. In this study, the daily celeration was calculated during baseline and practice, and the weekly celeration was calculated during maintenance as performance was assessed across weeks, not days. Bounce quantifies the amount of variability present in the data and produces a ratio to express its magnitude. The level change multiplier produces a ratio showing how much average performance changed from one phase to another (e.g., baseline to intervention). For all the ratios calculated, the multiplication (x) or division ( ÷) sign was affixed to indicate an increase or decrease across time (Kubina & Yurich, 2012). For example, a × 2.00 weekly celeration increase would indicate an increase of 100% per week, while a ÷ 2.00 celeration decrease would show a 50% reduction in performance. The only exception was Bounce, which is always reported using a multiplication symbol. All metrics were reported using the PT conventions described above. However, for ease of interpretation, the level change multiplier was transformed into a percentage to allow readers to evaluate the change in average performance more easily.

In addition to these metrics, two effect sizes were calculated, namely the Non-Overlap of All Pairs (NAP) and the Baseline-Corrected TAU (BCT). The NAP is an appropriate effect size measure for single case research with high correlations with the R^2^ effect size index (Parker & Vannest, 2009). The BCT assesses for the presence of monotonic trend in baseline and corrects it, if needed (Tarlow, 2017). We decided to use two measures as there is ongoing debate on which effect size is more appropriate for single case design research (Campbell, 2004; Lenz, 2013). These effect sizes were used to calculate the effect of the intervention on the performance of the participants in the experimental group and were calculated only for participants’ correct digits. Specifically, component skill effect sizes were calculated by comparing baseline to intervention data, while composite skill effect sizes were calculated by comparing baseline to maintenance data. Small effects were between 0 and 0.65, medium effects were between 0.66 and 0.92, and large effects were between 0.93 and 1.0. Finally, to compare the two group’s performance at pre- and post-intervention, a Mann–Whitney U test was conducted with Monte Carlo simulations drawing 10,000 samples to calculate the 99.9% confidence interval for the p-value.

## Results

### Component Skills

**Fig. 2:**
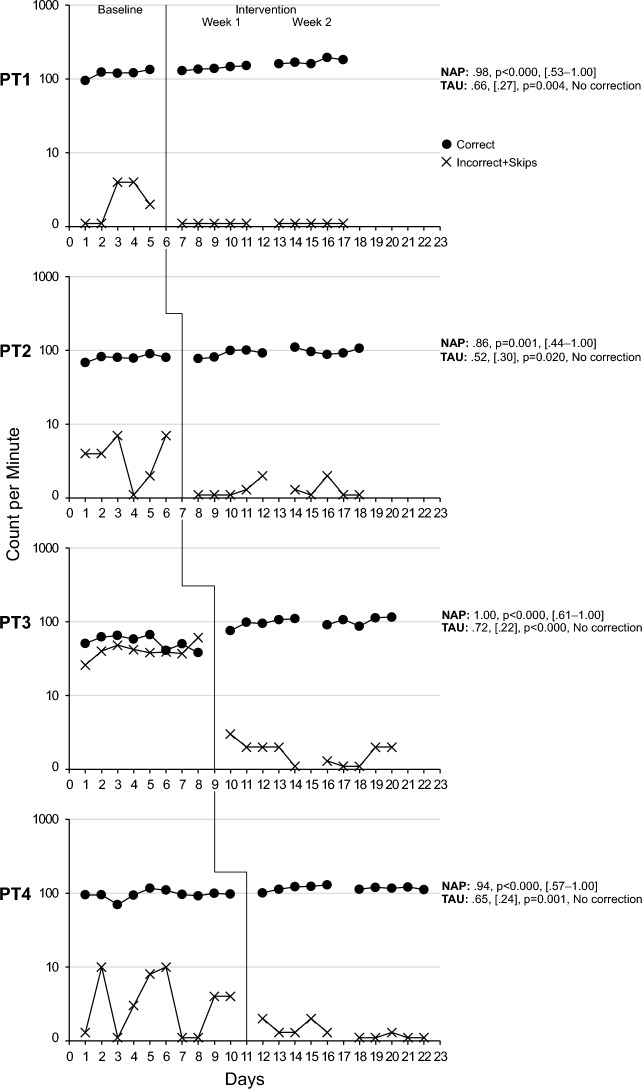
See-Say Number 0–20 (Component 1)

**Fig. 3:**
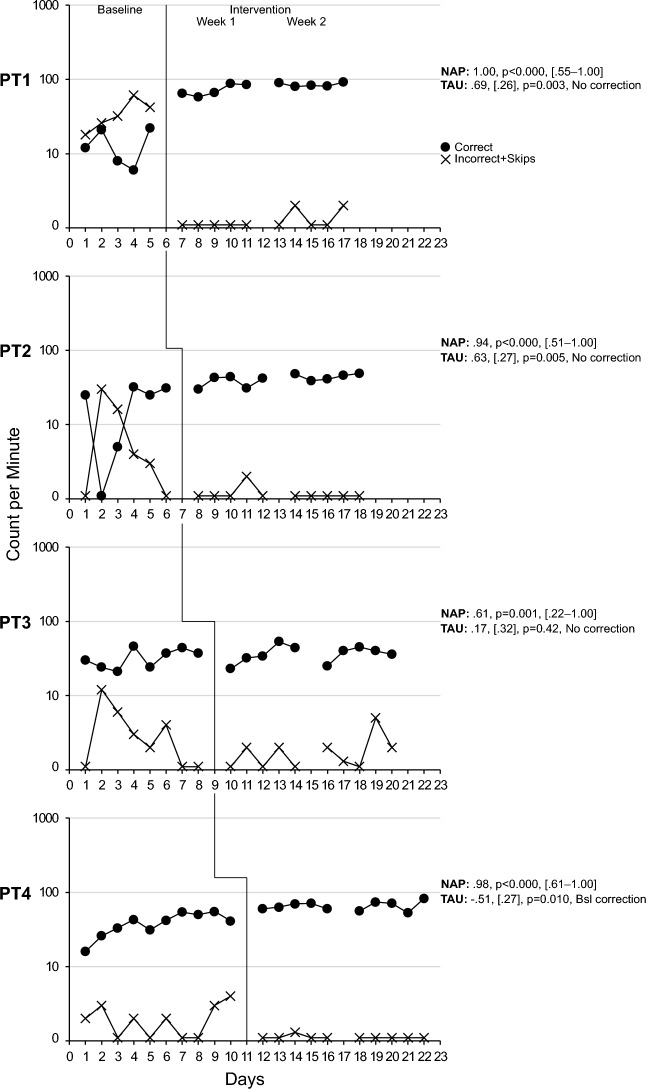
See-Say Counts three numbers (upward or downward; Component 2)

All Precision Teaching participants improved their performance following the introduction of PT and fluency training, albeit with a varying magnitude. Specifically, based on the level change multiplier, PT1 improved on average by 32% in component skill 1 increasing their performance from an average of 118 correct digits/min during baseline to 155/min during the intervention. They improved by 541% in component skill 2, increasing their performance from an average of 12 correct digits/min during baseline to 78/min during the intervention. PT1 also dropped below an average of 1 incorrect digit/min. An examination of NAP and BCT values shows that the intervention produced medium to large effect sizes (see Figs. 2 and 3, Tier 1). Their learning rate, as quantified by celeration, was low at × 1.24 and × 1.22, respectively, while bounce was minimal at × 1.10 and × 1.40, respectively, suggesting a stable performance throughout the two weeks of practice. PT2 improved on average by 18% in skill 1 increasing their performance from 79 correct digits/min to 94/min. They improved by 279% in skill 2, increasing their performance from 11 correct digits/min to 41/min, while producing small to large effect sizes (see Figs. 2 and 3, Tier 2). PT2 also dropped below an average of 1 incorrect digit/min. Their learning rate was low at × 1.10 and × 1.19, respectively, while bounce was minimal at × 1.30 and × 1.50, respectively. PT3 improved by 87% in skill 1 increasing their performance from 53/min to 99/min and by 14% in skill 2 increasing their performance from 32/min to 36/min while producing medium to large effect sizes for skill 1 and small effect sizes for skill 2 (see Figs. 2 and 3, Tier 3). PT3 also dropped below an average of 2 incorrect digit/min. Their learning rate was low at × 1.12 and × 1.15, respectively, while their bounce was minimal at × 1.40 and × 1.90, respectively. PT4 improved by 22% in skill 1 increasing from 96/min to 117/min and 78% in skill 2 increasing from 37/min to 66/min while producing medium to high effect sizes for skill 1 and medium to negative, and non-significant, effect sizes for skill 2 (see Figs. 2 and 3, Tier 4). Regarding skill 2, the BCT detected a trend in baseline that was corrected. As a result, a negative effect size was produced. PT4 dropped below an average of 1 incorrect digit/min, demonstrated a low learning rate at × 1.02 and × 1.05, respectively, while bounce was minimal at × 1.20 and × 1.50, respectively. In summary, all participants demonstrated positive, yet varying, levels of improvement across the two component skills with a very low number of incorrect responses, low learning rates, and a stable performance.

### Composite Skill

**Fig. 4:**
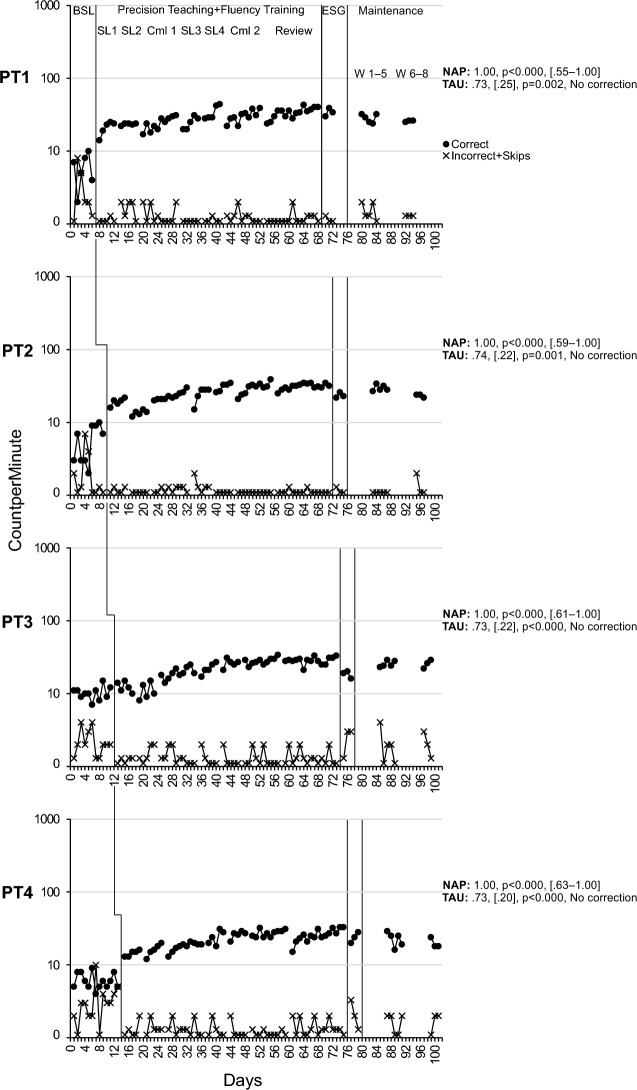
See-Say addition/subtraction facts (Composite)

All Precision Teaching participants made considerable improvements in this skill that produced medium to high effect sizes and were maintained well across time. Based on the level change multiplier, PT1 improved on average by 414% on the addition/subtraction facts increasing from an average of 5 correct digits/min in baseline to an average of 27/min during maintenance assessments, while dropping below an average of 2 incorrect digit/min in the maintenance assessments (see Fig. 4, Tier 1). Their average learning rate during practice, as quantified via celeration, was × 1.74 (range: × 1.10– × 2.58) suggesting a robust growth that at times exceeded the golden standard of a × 2 celeration (Johnson & Street, 2013). Their average bounce was minimal at × 1.30 (range: × 1.10– × 1.40) suggesting a stable performance across all practice slices. PT2 improved on average by 459% increasing from 5 correct digits/min in baseline to 27/min in maintenance, while dropping below an average of 1 incorrect digit/min (see Fig. 4, Tier 2). Their average learning rate was robust at × 1.84 (range: × 1.06– × 3.52), which also exceeded the × 2 expectation at times. Their average bounce was low at × 1.37 (range: × 1.10– × 1.90). PT3 improved by 153% increasing from an average of 10 correct digits/min to 26/min, while dropping below an average of 2 incorrect digit/min (see Fig. 4, Tier 3). Their average celeration was × 1.39 (range: ÷ 1.51– × 2.15) suggesting an acceptable learning rate that exceeded × 2 at times. Their average bounce was × 1.43 (range: × 1.10– × 1.80) showing stable performance. PT4 improved by 257% increasing from an average of 6 correct digits/min to 21/min, while dropping below an average of 2 incorrect digit/min (see Fig. 4, Tier 4). Their average celeration was × 1.53 (range: × 1.10– × 2.32) suggesting a robust learning rate that exceeded the × 2 expectation at times. Their average bounce was also low at × 1.30 (range: × 1.10– × 1.60). In summary, all participants made improvements in the composite skill, with a considerable increase in their average performance, a drop in their incorrect responses, robust learning rates, and a stable performance.

At the end of the maintenance assessments the KTEA’s math fluency subtest was administered again to all participants to make possible the comparison of performance pre- and post-intervention. Recognizing that the sample size is small, at pre-intervention, Precision Teaching participants had significantly lower math fluency ability than control participants, z = -2.78, p = 0.003, 99.9% CI [0.001, 0.005], while there was no significant difference between the two groups at post-intervention, z = -0.93, p = 0.41, 99.9% CI [0.40, 0.43]. The PT participants demonstrated considerable improvements that were greater than those of the control participants (see Table 4). Notably, although all PT participants were below the 15^th^ percentile rank during the pre-intervention assessment, they were above the 65^th^ percentile rank during the post-assessment, demonstrating a considerable and significant improvement.

**Table 4:** Participants’ Scores on KTEA’s Math Fluency Subtest

Participants	Pre-Intervention Math Fluency Standard Score	Post-Intervention Math Fluency Standard Score	Pre-Intervention Percentile Rank	Post-Intervention Percentile Rank
PT-1^a^	84	108	14	70
PT-2	80	106	9	66
PT-3	84	109	14	73
PT-4	77	107	6	68
C-1^b^	91	99	27	47
C-2	92	102	30	55
C-3	113	110	81	75
C-4	105	103	63	58
C-5	106	104	66	61
C-6	123	129	94	97
C-7	110	111	75	77
C-8	99	96	47	39
C-9	86	91	18	27

KTEA-3 = Kaufman Test of Educational Achievement—Third Edition

^a^PT1 to PT4 are the participants that received the intervention. ^b^C-1 to C-9 are the participants that received TAU conditions

## Discussion

This study aimed to evaluate the effects of a Precision Teaching framework that was delivered, in an original manner, via teleconferencing and included untimed practice, timed practice, goal-setting, graphing, and a token economy. A component-composite analysis led to the identification of three skills that were targeted with two of them being pre-requisites to the primary skill. All Precision Teaching participants improved their performance across all skills. Improvements in the two component skills varied in magnitude as there were cases of small improvements and cases of large ones, while improvements in the composite skill were large for all of them as evidenced by their performance in the maintenance assessments and the KTEA’s post-assessment. Control participants failed to demonstrate similar improvements further enhancing the study’s internal validity. Therefore, this study adds to the existing literature demonstrating the impactful outcomes of PT when applied to typically developing students’ mathematical skills (Chiesa & Robertson, 2000; Greene et al., 2018; McTiernan et al., 2018; Strømgren et al., 2014).

The COVID-19 pandemic’s effect on education is currently being investigated. However, emerging evidence suggests that mathematics was an area that was significantly affected (Gore et al., 2021; Weidmann et al., 2021). Instructional approaches that could be delivered as supplementary instruction would be particularly important as they could help students catch-up and meet the expected performance standards. In addition, it would be important to deliver instruction not only in person but also online. That way students could receive instruction from their homes, which could potentially mitigate any barriers to accessing supplementary instruction in person. That is why the results of this study seem promising as it managed to produce considerable improvements while using technology that was already available to participants. Notably, in this study, students only needed a phone, tablet, or laptop and an internet connection as the software used during the practice sessions was available for free.

Educators have argued that integrating technology within education is a multifaceted process that requires the use of elements that have been individually tested and shown to produce beneficial outcomes (Alabdulaziz, 2021). Precision Teaching has been applied and researched for decades and it has consistently produced beneficial outcomes across various areas (Branch et al., 2018; Datchuk et al., 2015; Lydon et al., 2019; Sawyer et al., 2021; Vascelli et al., 2020). Despite its heterogeneous application, its critical features have been presented across the different studies and have led to accelerated outcomes for all students (Evans et al., 2021). Therefore, PT seems to be a system that could be readily adopted to an online delivery as its elements have already gathered encouraging evidence about their effectiveness. Moreover, as shown in this study, PT’s online delivery requires access to hardware and software that would be generally accessible to students in mainstream education.

This study also led to a series of additional findings. First, students who have fallen behind in mathematics might be lacking even the most basic skills. For example, PT3 improved by an average of 87% in saying the numbers. Similarly, PT1, PT2, and PT4 improved by an average of 541%, 279%, and 78%, respectively, in counting. This suggests that these students have been moved up the curriculum despite having not mastered some of the most essential skills. This is an important finding as it highlights that the need for sensitive and frequent progress monitoring to ensure that students are not impacted by cumulative dysfluency (McDowell & Keenan, 2001; McDowell et al., 2002). In other words, students who are not provided with adequate support to master essential prerequisite skills might be unable to benefit from more advanced instruction and therefore struggle to keep up with their peers.

Second, the results regarding participants’ learning rates were mixed. Precision Teachers place a heavy emphasis on quantifying and monitoring learning through celeration. That is why they typically set an expectation that learning should optimally be at a × 2 value or above per week (i.e., a 100% weekly improvement; Johnson & Street, 2013) or at least above the absolute minimum of × 1.30 per week (i.e., 30% weekly improvement). Students demonstrated low learning rates for the component skills and robust learning rates for the composite skill. Specifically, celeration was below the × 1.30 expectation for all participants across both component skills. As for the composite skill, PT1 and PT2 produced celeration values below × 1.30 in two slices, while they exceeded the × 2 expectation in three and two slices ,respectively, out of a total of seven. PT3 demonstrated lower learning rates as they fell below the × 1.30 expectation in five slices and only exceed the × 2 expectation in one slice, while PT4 fell below × 1.30 in three slices and exceeded × 2 in one slice out of a total of seven. This suggests that the instructional arrangements can be optimized further as years of practical experience have demonstrated that students can achieve a doubling of their performance each week (Johnson & Street, 2013). Potentially, the goal-setting procedure could have limited participants’ celeration. In this study, we used the personal best approach, which focuses on the performance frequencies produced by students (Martin & Elliot, 2016). However, the minimum celeration line is also used in Precision Teaching, where the focus is on setting expectations regarding the weekly celeration values produced by students (Johnson et al., 2021). However, to our knowledge, only one study has compared the two goal-setting procedures, and the results did not suggest any significant differences between them (Vostanis et al., 2021). One way to further improve the intervention’s instructional design would be to provide participants with varying activities that use more than one learning channel set. This strategy has been suggested to increase student motivation, attention, and ability to generalize their performance to novel situations (Kubina & Cooper, 2000). In this study, participants were working on the See-Say channel set. Although this is a primary channel widely used in education, it would have been helpful if other channel sets were used, such as the See-Write or Hear-Say. Learning channels are a concept that has not attracted much attention in the scholarly literature but could potentially be helpful for teachers who are designing their instruction, especially since they seem to be related (Vostanis et al., 2022). Another way to improve instruction would be by considering the stages of learning (Jimenez et al., 2021). In this study, Precision Teaching participants engaged in combined untimed and timed activities from the study’s initiation. Potentially, practice could have been divided into two levels. The first level could focus on providing practice to improve their accuracy of responding, which would be the acquisition stage. When participants demonstrated improvements at this level, then they could transition to the fluency stage and engage in timed practice (Jimenez et al., 2021). That way, students could receive a more staggered and gradual approach to their instruction.

On a similar note, the effect sizes produced varied. Specifically, NAP values were particularly encouraging as they demonstrated a large effect in most skills and across all four participants. However, the BCT values were of a lesser magnitude. This discrepancy can be explained by the different calculations procedures in the two effect sizes and has been noted in the PT literature before (Vostanis et al., 2022). Therefore, it is safe to conclude that the intervention had a positive effect but without a clear magnitude that should be investigated in future replications.

Regarding the mastery assessment, the results were to be expected. Precision Teaching participants did not manage to meet the performance criteria set within the time provided for practice. As a result, they were not able to perform as well during the assessments of endurance, stability, and generalization. Nevertheless, their performance was considerably better than in baseline and was maintained well. Optimally, they would be supported to reach the performance criteria set, which would indicate that their performance has achieved fluency (Fabrizio & Moors, 2003; Johnson & Street, 2013). The assessment of mastery can be a useful tool in the hands of teachers. Through this short assessment, they can gather information about the participants’ functional mastery of a skill. That way they can make an informed decision about whether the student should be provided with additional support or can progress to the next stage of the curriculum (Johnson & Street, 2013). Through this approach cumulative dysfluency is avoided and students experience success, while the instruction is tailored around their needs without having a significant impact on educational resources. In other words, it is an effective and efficient way to monitor progress and engage in dynamic and strategic decision-making (Fabrizio & Moors, 2003).

Another finding was that Precision Teaching participants’ performance was particularly consistent throughout the practice. Precision Teachers quantify variability through the bounce metric. In this case, bounce was particularly low as it never exceeded a × 2 value. This fact suggests that the structured nature of the PT framework can lead students to engage in consistent performance while gradually improving it. Optimally, we would hope to help students achieve high learning rates, across a gradually decreasing number of sessions, and with consistent performance across those sessions. Such a learning profile would suggest that students have become agile learners. In other words, they have learned how to learn more effectively and efficiently, which is considered an essential outcome in PT (Meyer et al., 2021).

### Limitations

This study had various limitations. First, participants were not randomly allocated to the experimental and control conditions. Precision Teaching participants were highlighted by their teachers as needing additional support in mathematics. Moreover, assessors were not blinded to the study’s aims, which could have introduced bias during the assessments. Second, improvement in the component skills was not considerable in all cases. In some cases, participants made small gains, which suggests that the skill was already in their repertoire. In clinical practice, a different skill would have been chosen. However, this decision was not made in this project to ensure that the study’s internal validity would not be affected. Also, the gains in the primary skill were considerable and clear for all Precision Teaching participants, which suggest that the study achieved its primary purpose. Third, participants were recruited if they had access to an internet connection and a phone, tablet, or laptop. Some students might not necessarily have access to such equipment, which would make their participation in the lessons impossible. Fourth, the Control participants only received Teaching as Usual in their school settings with no additional practice. The Control participants allowed us to guard against threats to internal validity, such as history or maturation. Potentially, if they had received supplementary instruction, they could have made similar gains as the Precision Teaching participants. However, this study aimed not to compare Precision Teaching to other approaches but rather demonstrate that it can be an effective and efficient framework delivered online to help students catch up. Finally, social validity information was not gathered from the participants due to Covid implications. Although parental and teacher anecdotal reports were positive, the lack of participant feedback is a limitation.

### Future Directions

This study adds to the existing PT literature on training mathematical skills of typically developing students. Although the results are encouraging, replications would be required with more participants and across different skills. It would also be interesting to examine if additional software would make the process more effective and efficient. Finally, future studies should consider the limitations described above and try to account for them. For example, it would be great to randomly allocate participants to experimental and control conditions or blind the assessors to minimize bias during assessments.

## Data Availability

This study has additional data stored in the FigShare data repository. Supplementary materials, including this study’s Standard Celeration Charts, and examples of the worksheets used, can be accessed at the FigShare data repository following this link: 10.6084/m9.figshare.c.6227358.v4.
